# Structural constraints and dynamics of bacterial cell wall architecture

**DOI:** 10.3389/fmicb.2015.00449

**Published:** 2015-05-08

**Authors:** Miguel A. de Pedro, Felipe Cava

**Affiliations:** ^1^Centro de Biología Molecular “Severo Ochoa" – Consejo Superior de Investigaciones Científicas, Universidad Autónoma de MadridMadrid, Spain; ^2^Laboratory for Molecular Infection Medicine Sweden, Department of Molecular Biology, Umeå Center for Microbial Research, Umeå University, UmeåSweden

**Keywords:** cell wall, peptidoglycan, structure, cross-link, chain length, HPLC, muropeptides

## Abstract

The peptidoglycan wall (PG) is a unique structure which confers physical strength and defined shape to bacteria. It consists of a net-like macromolecule of peptide interlinked glycan chains overlying the cell membrane. The structure and layout of the PG dictates that the wall has to be continuously modified as bacteria go through division, morphological differentiation, and adaptive responses. The PG is poorly known in structural terms. However, to understand morphogenesis a precise knowledge of glycan strand arrangement and of local effects of the different kinds of subunits is essential. The scarcity of data led to a conception of the PG as a regular, highly ordered structure which strongly influenced growth models. Here, we review the structure of the PG to define a more realistic conceptual framework. We discuss the consequences of the plasticity of murein architecture in morphogenesis and try to define a set of minimal structural constraints that must be fulfilled by any model to be compatible with present day information.

## Introduction

The bacterial cell wall was first described as a region of specific staining behavior wrapping the cell body (Churchman, 1927). This structure was soon associated with the processes of cell division and growth (Knaysi, 1930, 1941). Introduction of electron microscopy in conjunction with the development of cell wall purification methods, demonstrated the ability of purified walls to retain cell shape (Salton and Horne, 1951a,b), and characterized cell walls as mesh bag-like macromolecules called sacculi (from the Latin for small bags, sing. sacculus; Weidel et al., 1960). These early techniques were also critical to define the glyco-peptidic nature of the cell wall, which became also known as the peptidoglycan (PG) layer, and led to the establishment of its polymeric nature (Salton, 1952, 1953, 1956, 1960; Weidel, 1960; Primosigh et al., 1961; Salton, 1961; Weidel and Pelzer, 1964). Schleifer and Kandler (1972) neatly summarized the knowledge on the structure of bacterial walls accumulated until the early 1970s and sort of wrote in stone most of the basic notions that still hold today, namely: the universality of the basic structural subunit; the long-glycan/short peptide idea; the chemical uniformity of PG and its planar, ordered, net-like organization in Gram-negative bacteria, among others. Unavoidably, some of these ideas derived into a geometric and static perception of the PG layer as depicted in so many articles and textbooks.

An aspect of PG studies important to stress at an early point is quantification. Most studies present the abundance of the individual kinds of PG subunits (muropeptides) as a percentage of the total number because this makes measurements independent of the absolute amount of sample. However, this practice makes it easy to underestimate the relevance of minor components. The amount of PG in an *Escherichia coli* cell is around 3.5 × 10^6^ monomeric units (Wientjes et al., 1991). Therefore, values as low as 0.1% mean that each cell has about 3 × 10^3^ molecules of that particular muropeptide, a meaningful number on biological terms.

Here, we will review the experimental evidence accumulated on PG structure and metabolism in an attempt to define a series of basic structural constraints that can help us to revisit current models of the bacterial cell wall. In this study we will center on the features of Gram-negatives because of the relative abundance of studies, and the (apparently) simpler organization of their sacculi. However, most of the information available comes from a very limited number of bacterial species and generalizations have to be taken with great precaution. Particular emphasis will be made on the influence of individual muropeptides on local structure and on how subtle changes in the subunits might influence the properties of the cell wall.

## The Structure of the PG Monomers and Linear Polymers

The primary structure of PG monomers was defined in the early 1960s and it was soon found out that most Gram-negatives shared as common basic subunit the disaccharide pentapeptide: GlcNAc-(β,1→4)MurNAc-(L)ala-(D)glu-(γ)-(*meso*)Dap-(D)ala-(D)ala (M5; Schleifer and Kandler, 1972). The monomeric subunit might suffer a number of chemical modifications, but in most instances, the alternancy of L- and D-amino acids, as well as the presence of a terminal D-ala-D-ala dipeptide is respected (Vollmer, 2008; Vollmer et al., 2008).

**FIGURE 1:**
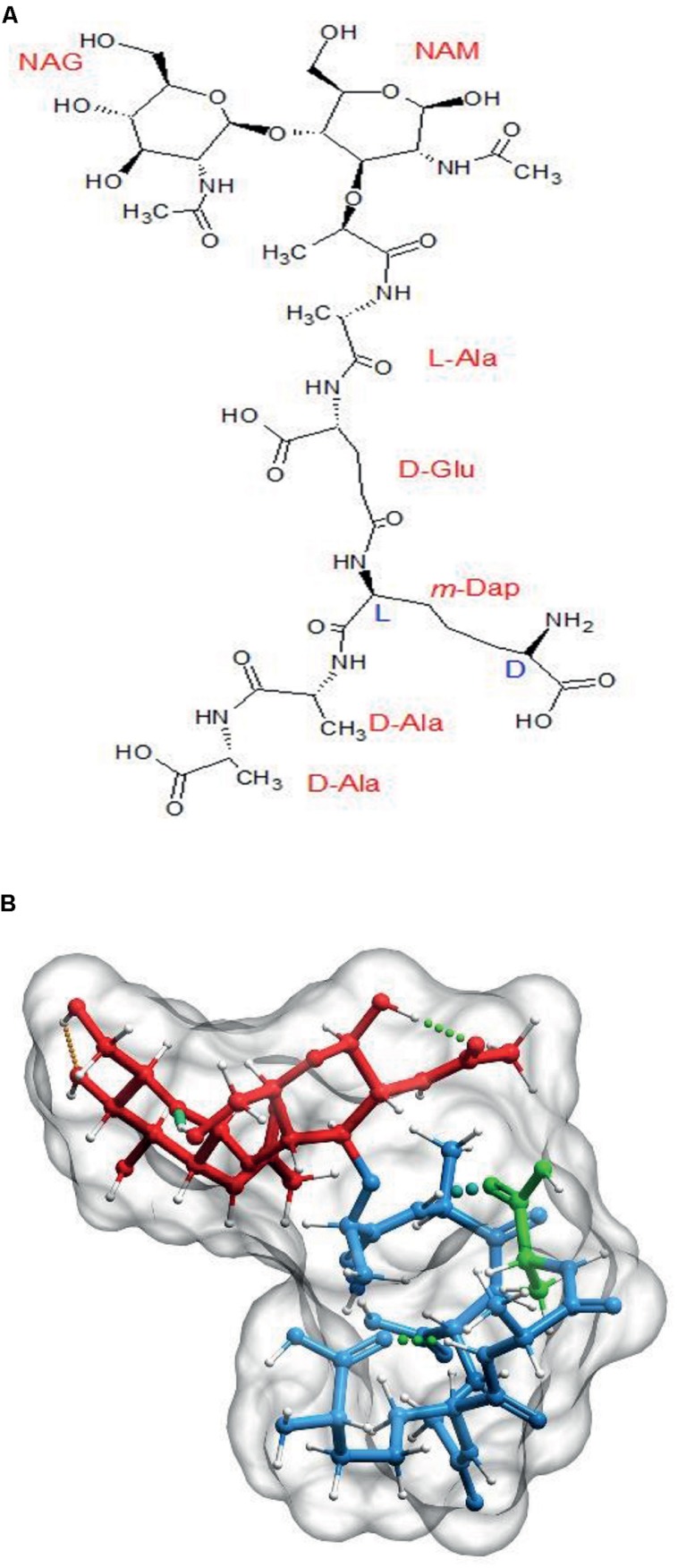
**The basic PG subunit. (A)** Explicit formula for *N*-acetyl-glucosaminyl-(β,1→4)-*N*-acetyl-muramyl-L-Alanyl-D-Glutaminyl(γ)-L*-(meso)*diaminopimelyl-D-alanyl-D-alanine (disaccharide–pentapeptide or M5). **(B)** Computer generated 3D representation of the same molecule after energy optimization. Red signals the glycan part, blue the stem peptide and green the terminal D-ala residue. Molecules were generated with Chemsketch (http://www.acdlabs.com/resources/freeware/chemsketch/), subjected to geometry and molecular mechanics optimization with Avogadro software (http://avogadro.cc/) using the MMFF94s field force, the steepest descent algorithm and a convergence of 10^-7^. Muropeptides were modeled in the absence of solvent. The optimized molecular structure was visualized and prepared for publication with Molsoft ICM software (http://www.molsoft.com/).

The PG monomer is a molecule with rather unusual biochemical and structural properties (**Figure 1A**) that, according to molecular modeling studies, results in the peptide moiety adopting a curled, compact disposition with the terminal D-ala interacting with the L-ala at position 2 by means of hydrogen bonds (**Figure 1B**; Barnickel et al., 1979; Meroueh et al., 2006; Gumbart et al., 2014). This disposition of the amino acid residues means that the stem peptides can potentially span considerably larger distances, from *ca.* 10 Å up to *ca.* 25 Å when stretched (up to 2.5 fold), as predicted by molecular modeling (Barnikel et al., 1983).

**FIGURE 2:**
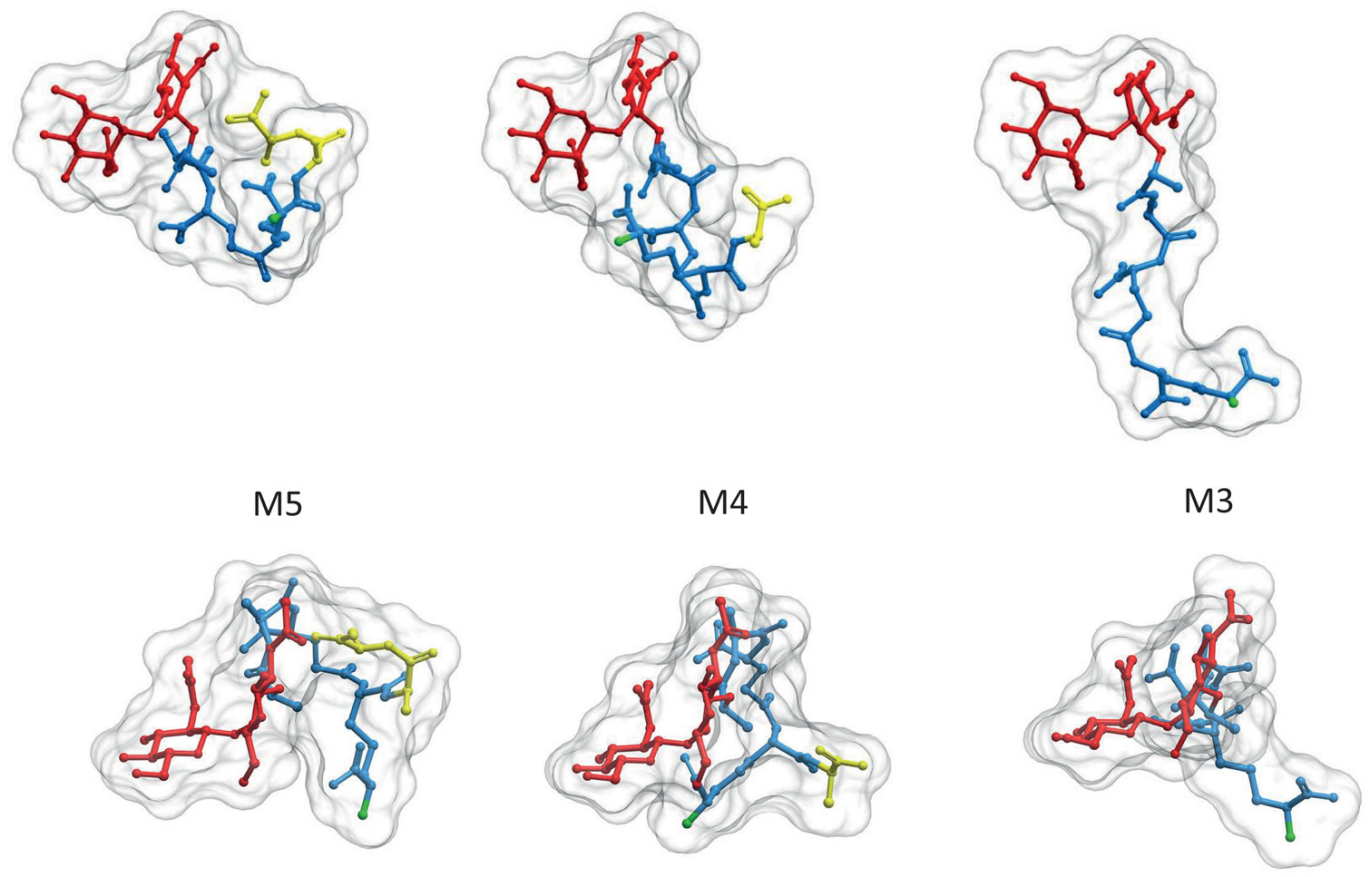
**Elimination of the terminal D-ala residue has strong influence on the 3D structure of PG subunits**. PG monomeric subunits derived from the M5 by elimination of the terminal D-ala (M4) or the terminal D-ala-D-ala (M3) are compared in two orientations, transversal to (upper panel) and from above (lower panel) the surface of the sacculus. To facilitate comparison muropeptides were rotated until GlcNAc residues overlap. Red designates the glycan moiety and blue the stem peptide. Terminal D-ala residues are highlighted in yellow. The 3D structures were calculates as in **Figure 1**. Hydrogen atoms were removed in the image, but considered for the calculation of the molecular surface.

Although some species as *Caulobacter crescentus* retain the terminal D-ala-D-ala in a high proportion of PG subunits (Takacs et al., 2010), most species contain mostly tetrapeptides and tripeptides following the sequential elimination of the D-ala residues to variable extents (Quintela et al., 1995). The kinetics of elimination of the terminal D-ala have been measured in *E. coli* (Glauner and Höltje, 1990). Assuming every monomer is added as a pentapeptide, nascent PG accumulated for as little as 20 s exhibits only about 25% pentapeptides, 60 s later it drops to about 5% and in a further few minutes goes well below 0.5%. As the terminal D-ala is required for the polymerization of PG the modified muropeptides are disabled to act as donors in DD-transpeptidation reactions (see below). Elimination of the terminal D-ala also affects folding of the stem peptides favoring a more extended conformation and displacements of the potential NH_2_ acceptor sites in the *(meso)*DAP residue (**Figure 2**).

In order to weave the polymeric cell wall the subunits must first form linear polymers, a reaction performed by bifunctional DD-transpeptidase-transglycosylase enzymes, the Class A Penicillin-Binding Proteins (PBP’s; Typas et al., 2012). The polymerization reaction happens in the periplasmic space and occurs by the transfer of a molecule of the activated precursor M5-P-Bactoprenol (lipid-II) to the C_4_-OH of the GlcNAc moiety in a second lipid-II molecule to generate a (β, 1→4) glycosidic bond (van Heijenoort, 2001). The growing glycan is then reiteratively transferred to new molecules of lipid-II causing longitudinal extension of the PG strand. The mechanism for the termination and release of newly made glycan strands remains unclear. In most bacteria, glycan strands display a molecule of (1→6) anhydro MurNAc (Anh-MurNAc) as the C1 terminal sugar, suggesting the participation of a transglycosylase activity in the process. However, there is no reason to discard the existence of alternative mechanisms. Indeed, *Agrobacterium tumefaciens* and *Mesorhizobium meliloti* either lack Anh-MurNAc or have amounts undetectable by HPLC and MALDI-MS techniques (Quintela et al., 1995; Brown et al., 2012).

**FIGURE 3:**
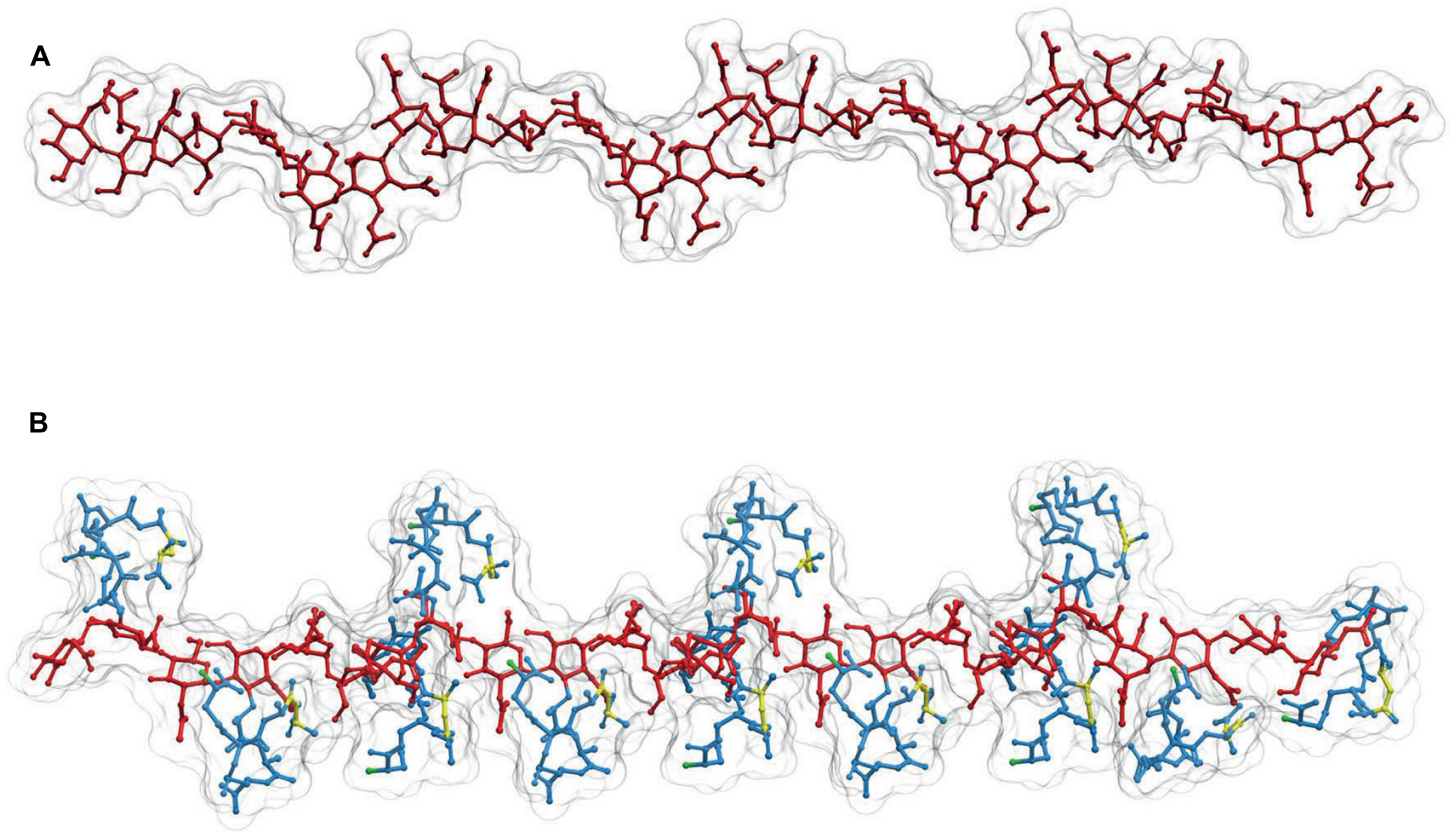
**Structure of linear PG polymers. (A)** Sugar backbone for a 12-mer linear PG polymer, with a helical pitch of three subunits/turn. The backbone is derived from the structure of **(B)** a PG strand made up of 12 M5 subunits. Red designates the glycan moiety and blue the stem peptides. The D-ala-D-ala dipeptides are highlighted in yellow, and the *(meso)*DAP free NH_2_ groups in green. The 3D structures were calculated as in **Figures 1** and **2**.

The sugar backbone of a PG linear polymer tends to adopt a right-handed helical structure (Leps et al., 1987; Meroueh et al., 2006; Gumbart et al., 2014). Calculations derived from actual NMR analysis of a linear dimer indicate three disaccharides per turn with consecutive subunits at roughly 120°, and a pitch of *ca.* 3 nm (**Figure 3B**; Meroueh et al., 2006; Gumbart et al., 2014) although a certain degree of variation in the helicity looks likely (Gumbart et al., 2014). This disposition implies that in a state of minimal energy, the sugar backbone is almost fully stretched, therefore when subjected to tensile forces the glycan polymer remains essentially unyielding. This fact contrasts with the relaxed disposition of the peptide moieties and makes the cell wall anisotropic respect to some properties (Gumbart et al., 2014). **Figure 3**, shows a likely conformation for a linear dodecamer as calculated by computer modeling. Other than the compact disposition of the stem peptides, is interesting to note that the critical regions, the D-ala-D-ala dipeptides and the D-NH_2_ groups of (*meso*)DAP molecules, involved in the all-important cross-linking reactions occupy rather accessible positions at the surface of the molecule (**Figure 3B**).

## Length of Glycan Strands

Length of the glycan strands is an important structural parameter of PG. Because the glycan backbone of PG strands is inextensible, the longer the strands the more rigid the structure of the sacculus. The PG can be compared to a composite material in that the global properties of the structure depend to a large extent on the length, orientation, and bonding of the fibers.

Because of the specific presence of Anh-MurNAc at the glycan ends (van Heijenoort, 2001), it is possible to calculate experimentally the average length (AL) of PG strands as the inverse of the molar fraction of Anh-MurNAc-containing subunits in the PG (Glauner, 1988). Available information supports a large variability in the AL of glycan strands amongst different bacteria. In *E. coli* the AL for cells actively growing in rich media is around 30 monomers/strand (M/S; Glauner et al., 1988). Most Gram-negatives analyzed so far fall in the 20–100 M/S range with a preference for the lower values (10–40 M/S). However, values lower than 10 M/S have been measured in *C. crescentus* and *Helicobacter pylori* (Costa et al., 1999; Takacs et al., 2010). AL is not constant but strongly influenced by the state of growth (Pisabarro et al., 1985; Lam et al., 2009; Cava et al., 2011), and by mutational or chemical alterations of cell wall metabolism (Romeis et al., 1991; Varma et al., 2007). In *E. coli* growth arrest causes a progressive reduction in AL (25–30%) until a constant value is reached in resting cells. Resumption of growth is accompanied by a progressive rise in AL which reaches its characteristic value after 2–3 doublings in mass. However, measurements of AL in newly made *vs.* total PG in *E. coli*, showed that AL was essentially constant for the new PG made during the transitions (Pisabarro et al., 1985). These observations support the idea that AL variations are not due to a change in the frequency of glycan chain terminating events, but rather to processing of macromolecular PG. Therefore, bacteria must have some modulatory mechanisms able to modify the AL of PG in response to environmental conditions.

An important aspect is that although the length of average PG strands (~10–100 nm) is very small when compared to the dimensions of the cell, in most cases the strands are considerably longer than the thickness of the sacculus, which is between 2.5 and 7 nm for hydrated sacculi (Yao et al., 1999).

Although AL certainly gives important structural information, it is the distribution of glycan chain lengths that really influences the properties of the sacculus. However, experimental determination of length distributions has been achieved in a very limited number of cases. The only extensive study was performed in *E. coli* (Harz et al., 1990). Whether or not the information obtained is representative for other bacteria is uncertain. The analysis showed a very wide and positively skewed size distribution with a modal value of eight M/S (Harz et al., 1990). However, this distribution only accounts for about 70% of the total PG. The remaining 30% could not be resolved into individual length classes but presents an AL equivalent of 45 M/S (Harz et al., 1990). At present, there are no data to choose a particular distribution of lengths for the longer fraction. However, as the shorter strands have to be at least 31 disaccharides long, and the AL is 45, the existence of extremely long (>500 disaccharides or roughly 500 nm) glycans is not very likely. The determination of both the AL and length distribution indicates that the sacculus is mostly made of rather short glycan strands but covering a very wide range of lengths, an aspect often neglected in cell wall models.

There have been a limited number of attempts to study how different conditions affect the glycan strand length distribution (Romeis et al., 1991; Obermann and Holtje, 1994; Ishidate et al., 1998; Ursinus et al., 2004). Particularly interesting is the comparison of regular rods and mini-cells in a mini-cell producing *E. coli* mutant (Obermann and Holtje, 1994). The results showed moderately shorter strands in the mini-cells with a reduction in AL from 28 to 25 M/S and a drift toward shorter lengths in the size distribution. A similar study performed with *E. coli* cell division mutants is also enlightening about local variations in glycan length distributions (Ishidate et al., 1998). This study showed that PG associated with the initiation of septation is likely made of relatively long glycan strands, and interestingly, the cell division protein FtsN requires strands >25 M/S to interact with septal PG (Ursinus et al., 2004).

Most of the information above is consistent with the existence of mechanisms able to make glycan strands of adequate lengths as a function of environmental conditions and surface topology.

## Cross-Linking of Glycan Strands

Formation of the sacculus requires glycan strands to be cross-linked to each other in a net-like fashion. Cross-linking is catalyzed by specific peptidyl-transferases which are able to transfer a peptide bond from a stem peptide (donor) to the free NH_2_ group at the D-center of the *(meso)*DAP residue of a second stem peptide (acceptor) from a nearby glycan strand. Most bacteria are capable of cross-linking reactions mediated by DD-transpeptidase activities residing in the bifunctional class A PBPs (Typas et al., 2012). These enzymes catalyze the transfer of the peptide bond linking the two terminal D-ala residues of the donor moiety, to generate a new peptide bond with a DD configuration. DD-transpeptidation seems to be a universal mechanism to interlink glycan strands, but it is not necessarily unique. Indeed, a few alternative cross-linking reactions have been described (Vasstrand et al., 1982; Glauner et al., 1988; Boniface et al., 2009; Bui et al., 2009). The more extended “accessory” cross-linking reaction is mediated by LD-peptidyl transferases (LD-TPases) which transfer the L–D peptide bond between the L-center of *(meso)*DAP and the D-ala at position 4 in the donor stem peptide to the NH_2_ group of the acceptor *(meso)*DAP (the very same as for the DD-reaction), generating a L-*(meso)*DAP →D-*(meso)*DAP peptide bond (Glauner and Höltje, 1990). LD-TPases are mono-functional enzymes, structurally unrelated to the PBPs, and are involved in a number of functions such as attachment of PG-bound lipoprotein (Magnet et al., 2007) and incorporation of D-amino acids (Cava et al., 2011). Although *E. coli* and most other Gram-negatives that have been analyzed have both kinds of cross-links, LD cross-linking is neither universal (Cava et al., 2011) nor essential for bacteria with both LD and DD cross-linkage (Magnet et al., 2008; Cava et al., 2011). The relative abundances of LD and DD cross-linking are very variable, but in some species can be equally abundant (Lavollay et al., 2008; Brown et al., 2012). Why some species have both kinds of cross-links is not clear yet. The fact that LD-enzymes are, in most cases, insensitive to beta-lactams has led to the proposal of LD-cross-links as contributors to escape antibiotic action (Gupta et al., 2010).

DD cross-linking of new strands is coupled to linear polymerization and is the reaction responsible for the incorporation of nascent strands into the sacculus. Indeed, analysis of nascent PG shows that right after incorporation, the new material already exhibits a content of DD-cross-links that is close to the average value for total PG (Glauner and Höltje, 1990). However, LD cross-links are initially absent (or below experimental detection limits) and then increase in a progressive way as PG ages (typically hours). This difference in kinetics supports the idea that LD cross-links are not involved primarily in the insertion of new precursors, but rather as part of PG maturation (Glauner and Höltje, 1990; Brown et al., 2012). However, LD-TPases involved in LD cross-linking work preferentially on those regions where incorporation of precursors occurs (Kuru et al., 2012; Siegrist et al., 2013), suggesting a close association between PG synthesis and LD-TPase activity. These considerations suggest that LD cross-links are an accessory feature of PG and may play important physiological and structural functions, particularly in those species where they are more abundant (Cameron et al., 2014).

**FIGURE 4:**
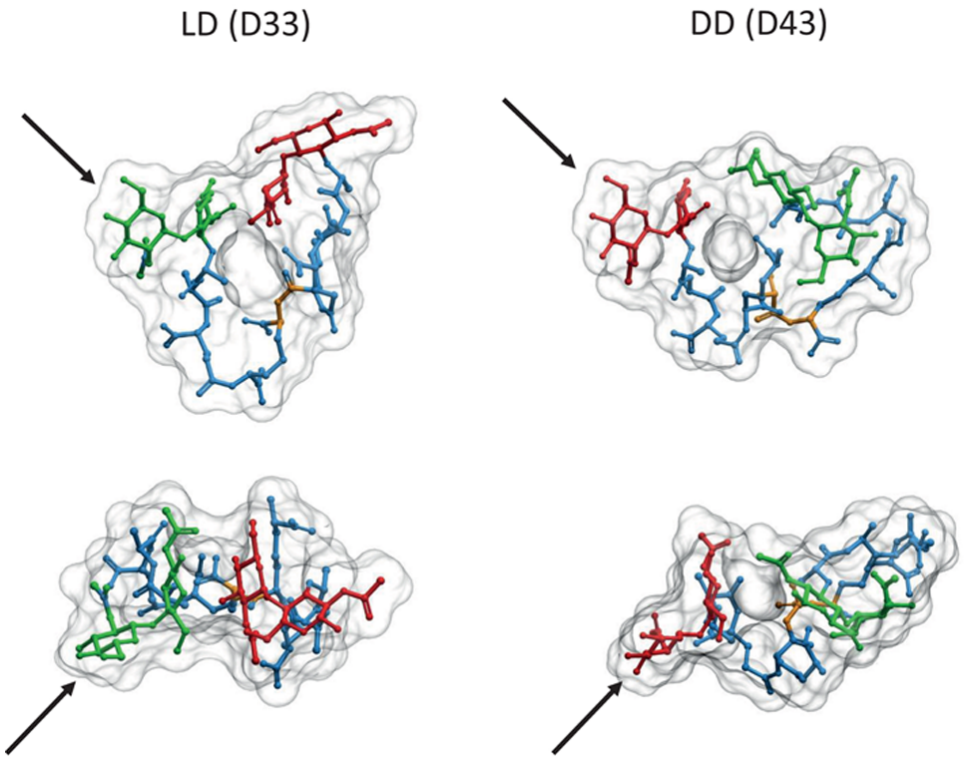
**Comparison of LD and DD cross-linked PG dimers**. 3D representations of molecules from the LD cross-linked *bis*-disaccharide-tripeptide (D33), and the DD cross-linked disaccharide-tetrapeptide-disaccharide-tripeptide (D43) dimers were generated as described in **Figures 1** and **2**. Upper images correspond to a transversal view, and lower images to a view from above. Each pair was oriented according to the GlcNAc residues indicated by the black arrows. Red, green, and blue designate the disaccharide for the donor moiety, the disaccharide for the acceptor moiety and the stem peptide, respectively. Yellow highlights the position of the cross-link. Note the inverted orientation predicted for the donor and acceptor moieties.

Comparison of computer models suggests important differences between LD and DD cross-links. The absence of the D-ala residue linking the two *(meso)*DAP molecules in LD cross-links makes the stem peptide more rigid, with a more extended conformation reaching a longer distance than the equivalent DD cross-link (1 nm *vs.* 0.7 nm distance between the MurNAc-C3 carbons of each strand, respectively; **Figure 4**). The possibility of additional hydrogen bonds in the DD cross-links affects the preferred orientations of the disaccharide moieties, and favors a more compact folding of the stem peptides. These differences imply that each kind of cross-link might fit optimally into subtly different relative orientations and distances between the glycans, and that local accumulations of one or the other crosslink should influence the local properties of PG differently. The longer range of LD cross-links might be especially well suited to connect glycans which are already under stress, as expected from the apparently post-insertional nature of this kind of cross-linkage.

**FIGURE 5:**
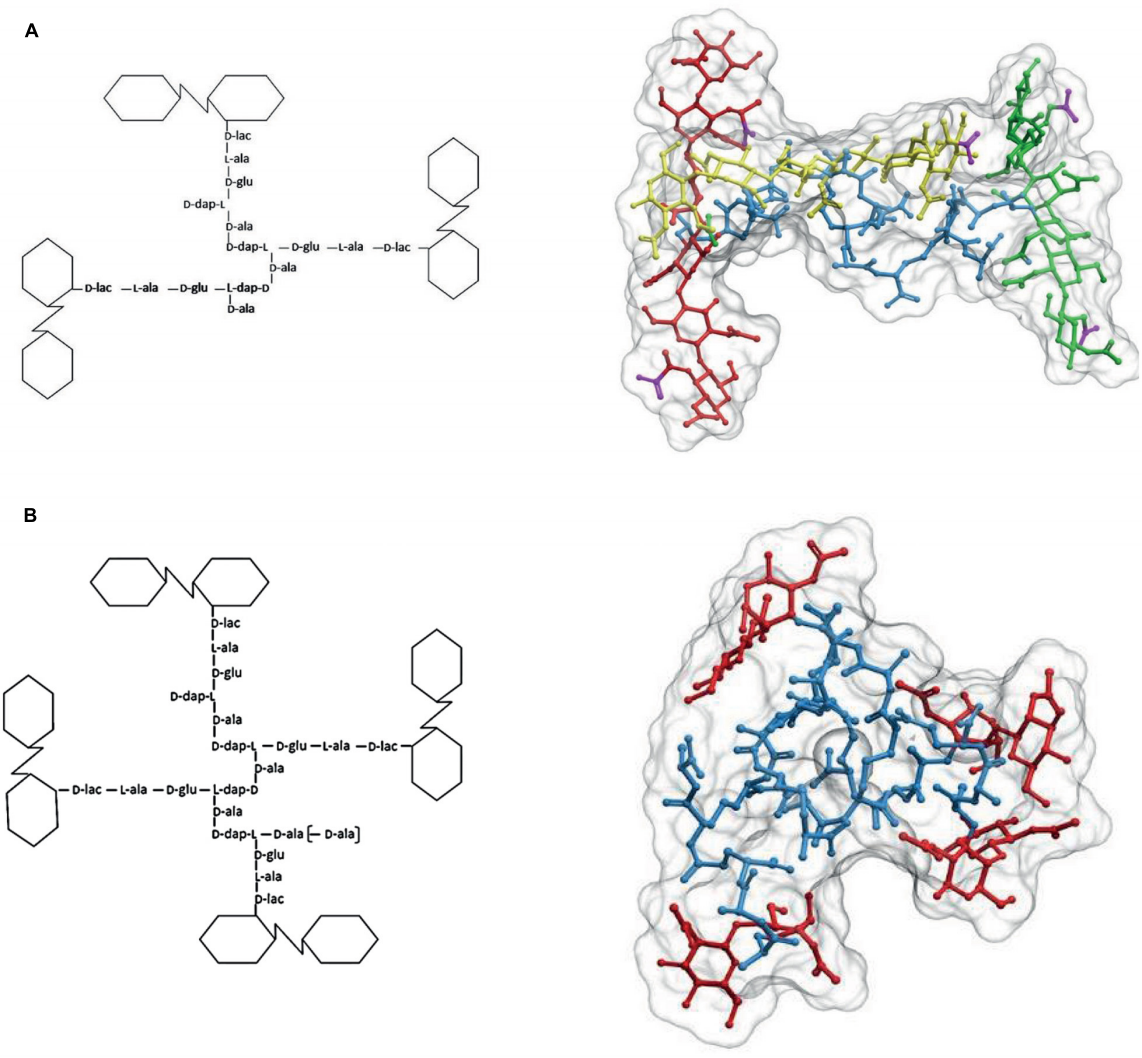
**The structure of cross-linked trimers and tetramers. (A)** Simplified formula and 3D representation of a molecule of the DD cross-linked trimer *tris*-disaccharide tetrapeptide. For a better visualization of the relative orientation of the strands, the glycans were extended by the addition of two disaccharides, one at each side, to each of the cross-linked moieties. Red, yellow, and green designate each of the glycan strands, and blue the peptide moiety. **(B)** Simplified formula and 3D representation of a molecule of the DD cross-linked tetramer *tetra*-disaccharide tetrapeptide. Red designates the glycan moieties and blue the stem peptides. The 3D structure was calculated as in **Figures 1** and **2**.

Cross-linking most frequently takes place between the stem peptides of muropeptides belonging to two glycan strands. However, in a variable but often considerable number of cases, three or, more rarely, four glycan strands become interconnected by their stem peptides generating cross-linked trimers and tetramers, respectively (**Figure 5**). Cross-linked trimers seem to be rather universal and have been detected in most Gram-negatives studied in relatively high proportions. Cross-linked tetramers are rarer than trimers. In most cases tetramers are not detected, but in a few instances sizable amounts have been measured, as in the case of *C. crescentus* (Takacs et al., 2010). Most abundant trimers are fully DD cross-linked. However, smaller amounts of hybrid (DD plus LD) and full LD cross-linked trimers are present in PG from bacteria able to LD cross-link PG (Glauner and Höltje, 1990; Brown et al., 2012).

According to Glauner and Höltje (1990), in *E. coli* DD trimers are readily detected in nascent PG with an age of 1 min. As the proportion of trimers in PG is rather low (about 3–4% of total muropeptides), this supports the idea of DD-trimers being synthesized concomitantly with the incorporation of new glycans into the pre-existing PG fabric. This is further supported by the kinetics of D-ala-D-ala elimination from nascent PG. Formation of DD-trimers should depend on the presence of subunits with a pentapeptide stem peptide in the donor moiety. The terminal D-ala is quickly removed from nascent PG. Therefore, DD-trimers should be made concomitantly or very shortly (<2 min) after incorporation of new subunits into a growing strand. With respect to how, when and where DD/LD hybrids or full LD trimers are made, there are no experimental data reported.

The presence of sizable proportions of cross-linked trimers and tetramers in the PG has deep structural consequences. As illustrated in **Figure 5A**, in the case of trimers the geometric constraints imposed by the nature of the molecule mean that the three glycan strands cross each other in a non-planar configuration. Indeed, at least one of the glycans has to follow a trajectory on a markedly different plane to that defined by the other two strands. A similar, even more complex situation is generated by the presence of tetramers (**Figure 5B**) which force a disposition of the strands in different planes. The normal abundance of trimers is rather variable, but taking *E. coli* as a reference (4–5% of total muropeptides) a sacculus would contain roughly 6 × 10^4^ such glycan-strand knots. As the number of strands per sacculus is *ca*. 1.1 × 10^5^ (3.5 × 10^6^ monomers per sacculus with an AL of *ca*. 30 M/S) (Harz et al., 1990; Wientjes et al., 1991) on average, each glycan chain participates in 1–2 of these knots. The contribution of tetramers is more difficult to estimate, as their presence in many species is doubtful. In *E. coli* their amount is close to the detection limit (0.2% of total muropeptides). This apparently low abundance still means about 2 × 10^3^ tetramers/sacculus, or that roughly one out of 16 strands is cross-linked by one tetramer. In those species were they are present in larger amounts as in *C. crescentus* (>3%; Takacs et al., 2010), their effect should lead to a very disordered orientation of the strands. Such a situation indicates that most strands weave over and under other strands and therefore, cannot form the kind of ordered, planar net often presented in representations and models of the cell wall.

## Knitting the Net, Cross-Links, and Glycan Strands

*Escherichia coli* PG has a considerable proportion (about 30%) of strands shorter than eight disaccharide subunits (DSs; Harz et al., 1990). The degree of cross-linkage is about 30% for *E. coli*. How this value is calculated means that there are 30 peptide bridges for each 100 muropeptides. Therefore, most strands equal or shorter than eight DS would statistically be cross-linked at two or fewer points. Such a structure would behave like a single long cross-link when subjected to stress (Costa et al., 1999), unless one of the cross-linking points were a trimer or tetramer connecting the short strand to two or three other strands, respectively. Quite interestingly, the terminal muropeptides of PG strands are hyper cross-linked (Glauner and Höltje, 1990; Lam et al., 2009; Takacs et al., 2010). Most strands terminate in a cross-linked dimer, but many in trimers and tetramers. The preference for a particular kind of terminal muropeptide could work as a compensating mechanism to ensure the continuity of the net-like structure under such circumstances. For similar reasons strands shorter than four DS (still about 10% of total PG) would be cross-linked at only one point and could not contribute to the strength of the stress bearing structure. These extremely short “strands” could represent material being recycled on its way out of the sacculus. Nevertheless, the above arguments assume a homogeneous distribution of cross-links, but this assumption could be wrong. An alternative would be that short chains were proportionally more cross-linked than long ones. Under these circumstances the short strands could play the role of staples joining longer chains at certain locations, whereas the long, under-cross linked, strands could deform more easily.

In some cases low AL values are associated with elevated proportions of cross-linked muropeptides, in particular trimers and tetramers (Takacs et al., 2010). The high proportions of trimers and tetramers might compensate for the shortness of the strands increasing the probability that they will cross-link to more than two other strands and therefore help create a relatively tight net made up of short strands. *H. pylori* represents an extreme case, with an AL of about 7 M/S in actively growing cells, but with a PG that lacks trimers and has a low degree of cross-linking (27% of dimers). In cases like this the NAc-muraminyl end of a short strand could be linked to the NAc-glucosaminyl end of another by a peptide cross-link, generating a longer linear polymer with alternating glycan and peptidic regions that could interconnect relatively distant strands and contribute to the strength of the sacculus (Costa et al., 1999).

## Organization and Orientation of PG Strands

Two important parameters define the structure of the sacculus: the total amount of PG subunits available, and the orientation of the glycan strands. Indeed, how PG is stretched to cover the area of the cell and how many PG layers exist in Gram-negatives have been matters of debate since the earliest works on cell walls (Wientjes et al., 1991).

Small-angle neutron diffraction experiments on purified *E. coli* sacculi (Labischinski et al., 1991) indicated that the thickness of PG in *E. coli* is non-homogeneous, with about one third of the surface thick enough (7 nm) to accommodate a triple layer of PG, whilst the rest has a thickness (2.5 nm) compatible with a single layered structure. However, the method only estimates the proportion of areas with each thickness, but gives no data about their distribution. These data are compatible with the distortions expected from the presence of cross-linked trimers and tetramers, as discussed above (Labischinski et al., 1991). Further support for a (partially) multilayered organization was obtained from cryo-EM (Hobot et al., 1984) and AFM (Yao et al., 1999). The later methodology provided the first measurements of PG thickness in the hydrated state and showed that hydration strongly influences this parameter. Direct determination of the amount of PG per cell (3.5 ± 0.6 × 10^6^ monomers/cell) and per unit of cell surface area (PG density) was also compatible with the kind of partially multilayered ordering suggested by other techniques (Wientjes et al., 1991). However, the calculations were based on a rather primitive model for the PG network (Oldmixon et al., 1974), and ignored the size distribution of strands and its possible relaxation effects on the PG fabric (Huang et al., 2012).

A second very important, and often neglected, observation from the AFM work was the demonstration that purified sacculi from Gram-negatives could have different thicknesses. Indeed, sacculi from *Pseudomonas aeruginosa* were about half the thickness of those from *E. coli* (Yao et al., 1999). This result clearly indicates that PG thickness might be a variable parameter. Indeed, studies on the effects of specific mutations (Lam et al., 2009), partial starvation for PG precursors (Prats and de Pedro, 1989) and the effects of D-amino acids on the structure of PG (Caparros et al., 1994; Cava et al., 2011) indicate that, at least some bacteria can accommodate large variations in the amount of PG per unit of cell surface, without apparent consequences for morphology, growth, and division. A relevant observation in *E. coli* is that the only significant variation detected in the composition of PG from cells with low PG content was a shortening in the AL of glycan strands (from 28 down to 19 M/S), a change that might change the elasticity of the sacculus to favor stretching (Huang et al., 2008). The surface density of PG seems to suffer large changes not only in response to harsh treatments, but also during the regular life cycle of bacteria (leaving aside those having specialized resistance forms), with resting cells accumulating considerably more PG per unit area than their actively growing counterparts (Caparros et al., 1994; Cava et al., 2011). Therefore, in at least some bacteria, the organization of the sacculus has to be compatible with drastic alterations in the PG surface density without causing macroscopic alteration in the shape and growth of the cell.

The relative orientation of the glycan strands with respect to the axes of the cell is another “hot topic” in cell wall biology. Because PG is not isotropic with respect to some characteristics, particularly elasticity (Gumbart et al., 2014), the disposition of strands in the sacculus has an important influence on cell wall properties. Measurements of the mechanical characteristics of sacculi show that they behave as a perfectly elastic material but with a high anisotropy (Yao et al., 1999). In fact, the elastic modulus for hydrated PG sacculi from either *P. aeruginosa* or *E. coli*, was 2–3 times larger in the longitudinal dimension (the long axis of the cell) than in the transverse direction, meaning that under pressure the sacculus would become proportionally longer. Such a behavior is a strong indication that the glycan strands transverse the long axis of the cell. In contrast, a perfectly (or close to perfect) perpendicular orientation of the glycan chains would be difficult to reconcile with arguments against a planar organization of these strands. Instead, analysis of purified sacculi by cryo-electron tomography supports a disordered circumferential arrangement of the glycan strands, which show a preferential, but not strict, transverse alignment relative to the cell axis (Gan et al., 2008). This disposition is also supported by advanced computational approaches, which find that such a layout best fits the mechanical properties of sacculi (Huang et al., 2008; Gumbart et al., 2014).

## Concluding Remarks

The arguments discussed above indicate that there is not such a thing as a “Gram-negative sacculus” and if there were, it would be quite far from a regular or highly ordered structure. Nonetheless, the most important point we wish to make is how little real structural information is available. Many readers will be surprised by the age of the citations in this review. The reason is simply that many important lines of work have been abandoned or not pursued with the intensity that is required to acquire more comprehensive information on cell wall biology. The biological diversity of the cell wall seems to be much larger than initially expected in terms of structure and composition, questioning the universality of the models developed on the basis of *E. coli* data. To explore the real range of cell wall structural variability an “omics” approach looks like the best direction to go. Recent methodological advances make fast analysis of large numbers of PG samples feasible, and open the way to a methodical, large scale survey of bacterial cell walls. New techniques in electron, atomic force, and optical (fluorescence) microscopy are also demonstrating enough resolving power to contribute substantially to the unraveling of the fine structure of cell sacculi. Further development and systematic application of these new capabilities should provide the data required for a realistic understanding of bacterial cell wall structure and biology.

## Conflict of Interest Statement

The authors declare that the research was conducted in the absence of any commercial or financial relationships that could be construed as a potential conflict of interest.
